# Design of a Wireless Sensor Network Platform for Tele-Homecare

**DOI:** 10.3390/s131217156

**Published:** 2013-12-12

**Authors:** Yu-Fang Chung, Chia-Hui Liu

**Affiliations:** 1 Department of Electrical Engineering, Tunghai University, Taichung 40704, Taiwan; 2 Department of Digital Literature and Arts, St. John's University, Taipei 25135, Taiwan; E-Mail: nancy@mail.sju.edu.tw

**Keywords:** telemedicine, home care, chronic diseases, ZigBee, physiological signal

## Abstract

The problem of an ageing population has become serious in the past few years as the degeneration of various physiological functions has resulted in distinct chronic diseases in the elderly. Most elderly are not willing to leave home for healthcare centers, but caring for patients at home eats up caregiver resources, and can overwhelm patients' families. Besides, a lot of chronic disease symptoms cause the elderly to visit hospitals frequently. Repeated examinations not only exhaust medical resources, but also waste patients' time and effort. To make matters worse, this healthcare system does not actually appear to be effective as expected. In response to these problems, a wireless remote home care system is designed in this study, where ZigBee is used to set up a wireless network for the users to take measurements anytime and anywhere. Using suitable measuring devices, users' physiological signals are measured, and their daily conditions are monitored by various sensors. Being transferred through ZigBee network, vital signs are analyzed in computers which deliver distinct alerts to remind the users and the family of possible emergencies. The system could be further combined with electric appliances to remotely control the users' environmental conditions. The environmental monitoring function can be activated to transmit in real time dynamic images of the cared to medical personnel through the video function when emergencies occur. Meanwhile, in consideration of privacy, the video camera would be turned on only when it is necessary. The caregiver could adjust the angle of camera to a proper position and observe the current situation of the cared when a sensor on the cared or the environmental monitoring system detects exceptions. All physiological data are stored in the database for family enquiries or accurate diagnoses by medical personnel.

## 1. Introduction

### 1.1. Research Background and Motivation

As more and more people are getting married late or even staying single, together with a deteriorating economic situation, the fertility rate in Taiwan has been declining significantly over the past few years. Although the fertility rate increased slightly in 2011 due to the government promotion campaign, the fertility rate in the past few years has dropped down to a half of the rate ten years ago. Progress in medical technology and the availability of medical attention provided by National Health Insurance have increased the national life expectancy. This places a heavy burden on the working population who have to take care of the dependent population, including infants and the aged.

According to the statistics of Council for Economic Planning and Development, the proportion of aged population increased from 6% in 1990 to 11% in 2010, and was estimated to increase up to 39% in 2060. According to the definition of World Health Organization (WHO), a country with a population aged above 65 exceeding 7% of the total population is regarded as an ageing society. According to this criterion, Taiwan has been an ageing society since 1993, and is estimated to become an aged society in 2018 and a super aged society in 2025. Currently, about one out of eight people are above the age of 65 years. As Taiwan became an ageing society, the problems of the elderly became a major concern of the society. The elderly need healthcare and medical treatment to solve their physiological problems. In regards to healthcare, the elderly often cannot handle their daily routines and require assistance and care from others. However, the use of in-home foreign private nurses is time-consuming, and the quality of service they provide is questionable. Consequently, most families choose to take care of the elderly on their own, but the family members cannot be with the elderly all the time and are lacking in professional healthcare knowledge and attention so emergencies cannot be handled properly as they are not aware of the physical differences and needs of the elderly. Consequently, emergency rescuers are not able to respond properly when an emergency occurs. Doctors also often have diagnosis difficulties because the elderly patients cannot clearly state their health problems. Doctors therefore need more complete information to achieve correct diagnosis. In this case, the need for the development of a healthcare system for nursing and treating the elderly has gradually been emphasized.

Nursing ocurrs at the beginning of healthcare, when the physiological conditions of the elderly are frequently examined and recorded in the database through wired equipment or manpower. Such data could not be measured continuously for 24 h, but only measured at certain time periods or when the elderly is physically uncomfortable. Bad physical conditions of the elderly are noted by the elderly themselves or by the facial expressions and exceptional behaviors, but the prime time for seeing a doctor is often missed. The promotion of health management has become a major policy for countries with these issues. Currently, in Taiwan there are up to 2.52 million elderly aged above 65, or about 10.9% of the total population. The long-term healthcare and nursing institutes for the elderly can merely accommodate some 60,000 people and only 43,000 people, or about 2% of the aged population, actually reside at such institutes, while the rest is taken care of at home. The demands for healthcare are increasing annually, while the homecare manpower is obviously in short supply. Integrating information technology into homecare therefore could significantly reduce the cost of manpower [1,2]. Besides, the healthcare services could be offered anytime through the system to reduce the probability of misreading the symptoms of the cared when nurses are not around.

### 1.2. Research Objective

As the population ages, more medical resources are required for expanding the healthcare in hospitals, homecare, and community care. Furthermore, the low fertility rate has resulted in insufficient human resources, so both domestic and foreign medical systems have introduced various wireless technologies and established healthcare systems to reduce the demands on medical personnel. A wireless network enables medical personnel to understand patient's physical conditions, and hence improves medical quality and reduces expenses. The wireless network technology allows mastering a patient's physical conditions, such as the physiological signals of human body temperature, heart rate, and electrocardiogram, and reducing the need for wires, so that the measuring equipment is not restricted to specific locations [1,3]. Nonetheless, a lot of healthcare systems overstress reducing medical manpower but ignore the friendliness factor so that the interface functions are incomplete, and the users find it difficult to use the systems. What is more, the construction and maintenance of some systems are so complicated that it costs a fortune.

This study aims to propose a wireless sensor network platform based on ZigBee, an emerging wireless transmission technology, to avoid negligence in taking measurements, to provide more complete healthcare, and to improve the quality of medical care for the aged population. Five objectives are covered in the healthcare platform:

(1) Allow the elderly to perform the measurements through wireless transmission without being restricted to specific locations, reduce the need for wires, and send out alerts to the medical personnel.
(2) Connect with the database through a system interface so as to query the previously measured data and display line graphs, with which the elderly could better understand the data and the family could directly see understand the past physical conditions of the elderly and interact with the elderly.
(3) Provide emergency alerts for the users to identify an exception physical conditions in time so that the elderly can receive the appropriate treatment.
(4) Store long-term measurement data in the database so as to inform the medical personnel about the past physical conditions; such measurement data could enhance the quality of diagnoses.
(5) Use a camera to transmit the user's image to the computer-based system so that the family could remotely observe the current conditions of the user.

This system could instantaneously transmit data and related analyses through the wireless network, through which alerts can be sent out and data can be stored into the database. The feature of automatic measurement with simple operation could reduce the costs of manpower and resources, allowing the family to realize the conditions of the elderly, while promoting the quality of healthcare, and reducing the burden on caregivers.

## 2. Related Work

### 2.1. Tele-Homecare System

The globally growing aged population has generated the increasing demands for medical services and long-term healthcare. The reality of insufficient medical human resources has encouraged a lot of research and development in the field of tele-homecare. Tele-homecare could enhance the abilities of the caregivers in a family and improve the quality of life of the elderly. The use of wire and wireless transmission communication, physiological signal sensors, and convenient two-directional interaction models with professional nurses [4–6] could enhance the freedom of the cared people and the autonomic management of illnesses, satisfy the healthcare needs of the elderly with chronic diseases, and reduce hospital expenses and the time and transportation expenses of travelling back and forth to hospitals.

There are more than 25 tele-healthcare system manufacturers in the US. The four major manufacturers—Honeywell HomMed, Health Hero, Cardiocom, and Phillips—cover about 40% of the entire market. Honeywell HomMed provides the users with needed options according to the budget of the user, and features straightforward management of the medical environment for patients and enhanced graphic data of patients. Health Hero, taken over by Bosch in 2007, offers the Health Buddy System to provide an advanced user interface with real-time analyses of health information. The elderly, the family, or the nurses can connect via the Internet using mobile phones, personal digital assistant (PDA), and personal computers to query the physiological data of the elderly, which could provide doctors or nurses access for data retrieval, management, and analyses. Cardiocom, a clinic remote medical service supplier, started to provide telemedicine systems, tele-monitoring equipment, and telemedicine services to reduce unnecessary hospitalization in 1999. Phillips, a multinational enterprise, offers lighting products, audiovisual equipment, and healthcare products and actively invests in medical businesses. It acquired Respironics, which used to be the leading manufacturer in sleep therapy and respiratory health in the world, in 2007. The respiratory monitoring products developed by Respironics have reinforced the status of Phillips in the medical industry. Moreover, the tele-medical services of Phillips contain heart monitoring functions which can detect symptoms like cardiac dysrhythmia. Such healthcare systems inform the doctors of emergencies and allow patients to read their own health reports. Phillips has successfully completed several merger deals with other manufactures in the homecare industry, such as XIMIS, Witt Biomedical, and Lifeline. Its homecare department has produced a series of non-hospital home treatment and monitoring products, benefiting about a million high-risk elderly patients in the US and Canada. Patients at home or in nursing institutions are taken care of by the medical facilities of Phillips. Various enterprises are actively developing more individualized healthcare systems. Apparently, the tele-healthcare industry has become a gold mine for international manufacturers. National tele-healthcare services are practiced at about 100 places in Japan, in which more than 90% are administered by local governments for the tele-physiological monitoring and emergency rescue of the elderly. NEC, Toshiba, Sanyo, and Panasonic are the major participants. Current telemedicine systems can exchange health data and information, including two-directional interactive image and voice for remotely diagnosing illnesses.

### 2.2. Wireless Tele-Homecare System

Aiming at the elderly in a family, a tele-homecare system receives the physiological information using wireless technologies and simple operation interfaces [7,8] for the elderly. The systems aim to reduce the costs of manpower for checking on the basic health conditions of the elderly and monitor the elderly around the clock to reduce accidents. When it is applied in the nursing station in a hospital or nursing care centers, the measured human body temperature, frequency of measures, and exceptional reminders could be adjusted according to the demands.

The integration of temperature sensor and wireless technologies could replace manpower for measuring human body temperatures. Besides, e-measurement allows increasing frequency of measurements which decreases accidents and stores the human body temperature in the database on a server for nurses monitoring the temperature changes of the cared people [5]. The development of tele-homecare systems enables self-measurement of physiological conditions by the elderly at home, without visiting hospitals. The system could assist in recording and analyzing so that the medical personnel understand the physiological changes of the elderly [9–11]. The degradation of various physiological functions would result in many exceptional physiological conditions of the elderly. When detecting slight physiological exceptions, the system would provide proper suggestions to assist the elderly patients. For serious exceptions, the system, in addition to offering suggestions and reminders to see a doctor, would automatically advise the caregivers or the family for necessary assistance [6,12]. In addition to the ability to store records and detect exceptional records in real time, the major merit of the tele-homecare system is that medical personnel or the family can acquire timely physiological information to take proper actions. The friendly interface of the system is helpful in reducing errors and allowing the users to set the alert ranges themselves.

Wireless networks are generally used for transmitting information in a tele-homecare system which removes the constraints of a wire-connected sensing device. The cared therefore could freely move around in a more convenient living environment. A lot of research has been conducted to improve the transmission efficacy of running tele-healthcare on a wireless sensor network (WSN) platform, such as using cluster architecture for power efficient transmission or utilizing smart agents for better resource management.

Furthermore, a number of tele-homecare systems have also been tested in some research experiments. For example, in an application of the sensor-based ZigBee technology in homecare, a wireless sensing device is placed in the neck of a patient to take his body temperature and the resulting reading is automatically transmitted to a server in real time. Visual Basic in Microsoft Visual Studio.NET 2003 was employed to develop an interface allowing the nurses to observe the temperature changes of the cared so that the nurses could take proper action as exceptions occur.

Radio frequency identification (RFID) was utilized for establishing a moveable login-in system in [13]. ZigBee was applied to transmitting the measured signals, by integrating various physiological measuring devices like thermometer, blood pressure gauge, and oximeter, to a personal computer for statistical analyses, data processing, and storage. When the measured signals of the cared exceed normal values, education and suggestions were provided to the cared in real-time, and e-mails and messages were automatically sent to the family and the caregiver.

A wireless sensing system was designed in [14], through which the physiological data of the cared were monitored so that the family and the medical personnel were informed in real time when there was an emergency. Both RFID and wireless sensors were utilized for monitoring the position and the physiological data of the cared, including body temperature, oxygen saturation, and respiratory rate.

### 2.3. Introduction and Comparison of Wireless Technologies

Various network technologies have been developed to meet specific application requirements. Common wireless networks include Worldwide Interoperability for Microwave Access (WiMAX), Bluetooth, Wireless Fidelity (Wi-Fi), and ZigBee [3,15,16]. An introduction and comparison of these various approaches is shown in Table 1.

(1) *WiMAX*. Also called the fourth-generation wireless communication (4G), it is used by mobile wireless broadband, which follows the IEEE 802.16 standards [17] and has a maximum transmission distance of 7–10 km and a maximum speed of 70 Mbps. It can replace fixed networks. It offers high transmission rates and broad communication coverage, but has not been very popular because of the high cost.
(2) *Bluetooth*. Bluetooth, applys the IEEE 802.15.1 standards [18] and is a short-distance wireless transmission technology mainly applied to wireless communication between mobile phones and hands-free devices as well as computers and external wireless peripherals, such as mice, headphones, microphones, and printers.
(3) *Wi-Fi*. Wi-Fi, following the standards of IEEE 802.11 [19], has a transmission rate of about 11 Mbps, and is a high-speed wireless network protocol widely utilized in the past years for network connections, such as laptops, smart phones, and tablet PCs. However, the power consumption is comparatively high.
(4) *ZigBee*. ZigBee was originally set up for home networks. Its continuous development now allows broader applications, in addition to home networks. The specifications follow the IEEE 802.15.4 standards [20], with a transmission rate of about 250 Kbps. Since ZigBee offers low power consumption, low costs, long communication distance, and multiple nodes, its applications could be broadened with various sensors.

### 2.4. Introduction to ZigBee

The wireless network technology ZigBee is utilized in this study. It is a short-distance wireless transmission technology that has been rapidly developed in the past years. It is established by the ZigBee Alliance and the IEEE802.15.4 Team along with several companies, and it stresses low costs and low power consumption. The rapid development of ZigBee has led to the inclusion of functions not restricted to general home networks, and it has also been introduced into industry and medicine. ZigBee, with low transmission rates (250 kbps), short distances (generally 50–100 m), and low power consumption, is easily installed and equipped and it supports a large number of network nodes and several network topologies. Moreover, it is fast, reliable, and safe with lower costs. The amount of transmissions of ZigBee is comparatively less as is often used for simple wireless control, including the simple applications for homes industries, and medicine, such as anti-intrusion sensors, lighting control, toxic gas (like carbon monoxide) detection, medical sensors, and patient emergency alarm calls [21]. It can facilitate medical treatment procedures by transmitting the measured signals anytime, in comparison with traditional wired equipment. The positioning function of wireless networks can be used to monitor the movement of patients and monitor the human body temperature or pulse anytime [22,23].

Among the different wireless technologies, the ZigBee Wireless Transmission Module is often selected for healthcare systems that require frequent measurements and text-based data transmission that the low power-consumption. Besides, in consideration of the simplicity in operation and user acceptance, ZigBee can be easily installed, has lower costs, and supports various networks. When a node is out of order, other nodes could be selected as the path for signal transmission to ensure the continuous operation of the entire system, which is critical for the life safety of the elderly. The advantages of ZigBee are further summarized as follows:

(1) Low cost. The simple protocol and small storage account for the low costs of ZigBee, so that it costs merely US $2 per chip.
(2) Low power consumption. ZigBee uses sleep mode when not working, and the startup time is merely 15 ms, which is faster than Bluetooth which takes 8 s to add a node. ZigBee can rapidly recover and connect when nodes are required and go into sleep mode again after transmitting data. Such a transformation allows ZigBee to prolong battery life. Various types of batteries could support ZigBee service for periods ranging from six months up to two years.
(3) High reliability. With Collision Avoidance of talk-when-ready, a confirmation reply would be sent after receiving each control instruction or transmitted data envelope feature. Without such a reply, the transmission would proceed again to ensure the transmission of physiological information. ZigBee thus presents high-reliability information transmission.
(4) High expandability. ZigBee can cover up to 65,536 nodes in the network. Such network nodes would automatically construct a mesh network for rapidly allocating nodes. In other words, each ZigBee node could be connected with many nodes to show the larger capacity.
(5) Support for various network structures. ZigBee supports star, tree, and web structures, which have distinct advantages for different projects. A web structure could provide higher reliability so that other nodes could be used as a path for transmitting signals when one node is broken.
(6) Security. ZigBee offers three levels of security mode, including non-security setting, utilizing Access Control List (ACL) for preventing illegal data acquisition, and applying 128 bit Advanced Encryption Standard (AES) with complete data transmission checking function. Such measures ensure the security of ZigBee and prevent the data from being changed or accessed by attackers.

## 3. Research Method

### 3.1. System Framework

**Figure 1.**
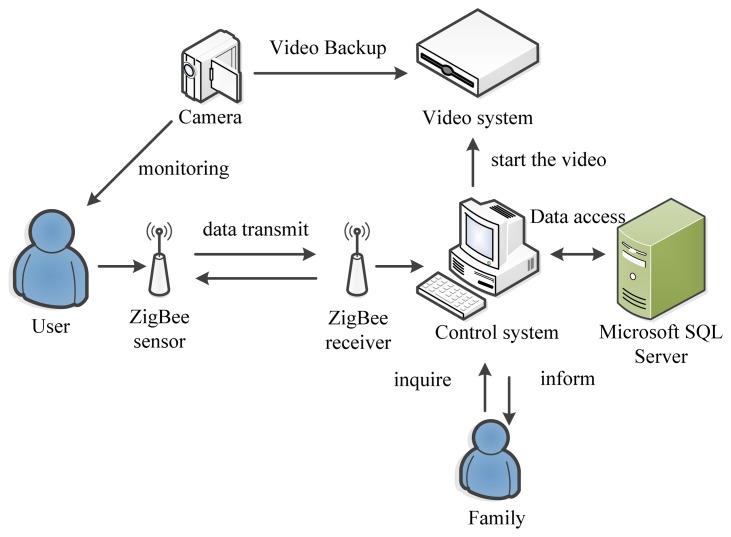
System framework.

Figure 1 shows the basic design framework for this system, which aims to monitor the physiological signals of the elderly. In this study, the temperature of human body is regarded as the major measurement data, and the physiological signals received by ZigBee are transmitted to a computer to be stored in a database or recorded on the interface. Users and their family would be automatically informed of exceptional signals so that they could query the recorded physiological signals through the computer-based system, and they also can observe the current situations through the video function of the system. The image at the user end would be immediately viewed by connecting the system with a camera.

The proposed system design contains the wireless transmission and the system interface. The wireless transmission proceeds in two parts: the first part is the connection between the sensors and ZigBee, and the second part is the programming codes required for the CC2530 main chip for the ZigBee algorithm; such programming codes could be directly transmitted back to the user for alerts to reduce the computation time. Visual Studio 2008 is used as the system interface of the computer display for analyzing the received data, judging the exceptional start, informing the user to start the video, and storing into a Microsoft SQL Server database.

### 3.2. System Procedure

**Figure 2.**
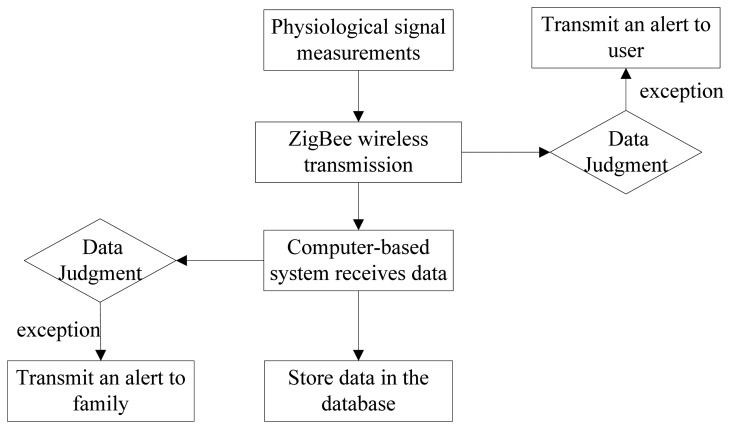
Measurement process.

While measuring following general conditions as shown as Figure 2, the user could transmit the signals to the computer-based system through the ZigBee wireless network after measuring the physical conditions. When physical discomfort is measured, the wireless network would transmit the exceptional data so that ZigBee would directly transmit an alert to inform the user through a buzzer. Not only does the user understand the personal health conditions, but ZigBee would also transmit the data back to the computer-based system for making a judgment to inform the family. The measuring time and the physiological data would be stored in the database.

When the doctor and the family intend to view the past physical conditions of the elderly, they could query the database, where the date and data type are selected on the interface. The mean of the daily data would be calculated and the trend diagram is shown on the interface for doctors making diagnoses.

### 3.3. System Function

**Figure 3.**
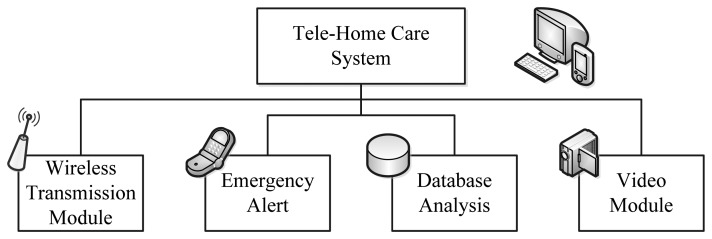
System functions.

In order to completely take care of the user's health conditions, the Wireless Transmission Module, Emergency Alert, Database Analysis, and Video Module functions are the essential elements for a healthcare system. The functions, as shown as Figure 3, are introduced as follows:

(1) Wireless Transmission Module. To reduce the costs of wires and to facilitate communications, the wireless technology ZigBee is utilized for transmitting the physiological signals measured to the computer-based system and for calculating the exception of such data for alerts.
(2) Emergency Alert. When exceptional data are transmitted, ZigBee would send an alert to the user and the computer-based system. Once the system determines such data is exceptional, it will transmit the data and time to the family through e-mail so that the family could realize the health conditions in time.
(3) Database Analysis. While transmitting data to the computer-based system through ZigBee, the system would retain the record of time and ID related to such data in Microsoft SQL Server. When the user intends to understand the physical conditions over a certain period, a time period and the queried data type could be selected on the interface. The system runs the mean of daily temperature and displays the graph on the interface so that the user could clearly observe the physical conditions.
(4) Video Module. When exceptional data appear, the video function of the system allows the family to observe the current conditions of the user through a camera. The monitoring host is equipped with video storage hardware for the user to view past images anytime.

#### 3.3.1. Software Design

In the study, the software Visual Studio 2008 was selected for establishing the display interface. Such a design allows the user to easily operate the system. The modules in ZigBee cannot perform multi-functional processing that the data are transmitted to the computer for processing, where the data are easily classified and stored into the database, and for future enquiries. Three major objectives are planned in the system, including measuring, sensing, and monitoring. The user measures the physiological signals and transmits the data through the Wireless Transmission Module to the system for data storage and analyses. Environmental sensors are placed in the user's living environment for measuring the basic elements of temperature, humidity, and brightness. The system is connected with video monitoring equipment so the user's real-time images could be observed while exceptions occur.

**Figure 4.**
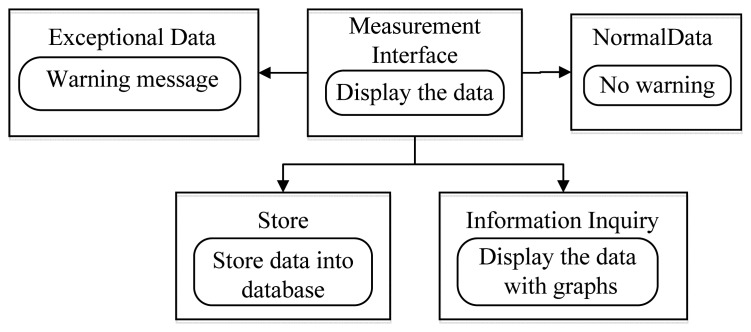
Display interface design procedure.

(1) Measuring data. In measuring data, the display interface appears in the shape of figure. Because the ZigBee Wireless Transmission Module and the computer are connected with by USB, an accurate COM Port needs to be selected for successfully receiving the data and the communication between the computer and ZigBee. Different display functions are also included, as shown as Figure 4. In this system, the measured data would be displayed in real time onto the interface, where keeps measured time and uploaded data. For monitoring, the recorded values in a normal range are displayed on the display interface. When the physiological signals are too high or too low, an alert window is started to remind the family. Meanwhile, the user would receive an alert from ZigBee. When exceptional data appear for a long period, the family would receive a notice through e-Mail directly, and all data and times would be stored in the database. When the user and the family perform a query, these data are calculated and illustrated with figures.
(2) Data storage. The data could be stored in Microsoft SQL Server in real time. The storage intervals are divided into normal and exceptional data. If the data appear normal, the interval would be extended, whereas the interval would be shortened as exceptional data appear. The data to store contains ID numbers, measured data, and measured time, like year, month, day, hour, minute, and second. ID numbers are used for recording personal physical conditions so that it will be more convenient for different data queries from several users.
(3) Data display. Two DateTimePickers are utilized for setting the time interval for the convenience of querying records. There is a query button; when the button is pressed, the mean of daily data are illustrated with a chart.
(4) Video function. A function is designed to start the video. When the button is pressed, the system would ask for a legal IP and password of the user's camera to directly connect to the user's camera. The program provides quad-split screens for viewing images from four different cameras.

## 4. System Implementation

### 4.1. Program Recording on the ZigBee Chip

The CC2530 chip in ZigBee is used for driving the entire system, and a program writer stand is equipped on the ZigBee chip for connecting with a program writer. The writing program offered by the IAR allows distinct writing according to the demands. ZigBee offers the function of transmitting the data from the measuring equipment to a computer-based system. In order to reduce the response time to emergency alerts, the data are calculated in the process. When the data exceed the normal range, such data would be sent back to the user with a buzzer, informing the user to realize the exceptional conditions. For example, when the measured data exceed the standard human body temperature, either too high or too low, the buzzer would remind the user and the exceptional data would be displayed.

### 4.2. Interface Design with Visual Studio 2008

The transmission settings are not discussed in the design as the ZigBee manufacturer offers the transmission program. After starting a computer-based system, a restart function is provided to cope with the problem of not finding a suitable COM port. The data can be immediately received after the successful connection. The sub-page for selecting the measured data is designed on the interface for viewing current data. Text-based alerts would be displayed when the data appear exceptional, and such data are stored in the database according to the time interval. Since the data are stored anytime, the quantity of data would be large. Besides, the temperature of human body should not change abruptly, so the time intervals are designed for normal and exceptional situations, as the user's physiological signals need to be frequently monitored when exceptional data appear. A column for indoor environment data is also designed on the interface. The ZigBee sensor chip in the room could monitor and transmit the temperature, humidity, and brightness to the computer-based system. The indoor environment could be further controlled by combining the system with various electric appliances. Moreover, a camera could be equipped in the user's room for the family viewing the current situation of the user. Chart is utilized for the enquiry graph, in which two DateTimePickers are offered for the user to select the time interval and query the data in the database. The mean of daily data is then calculated and represented in the shape of a figure.

### 4.3. Establishment of Microsoft SQL Server

The database is established for storing all measured data, allowing the user to query all records. The data integration can be enhanced by following certain steps to establish the database. The data correlation in the database is then enhanced for clearer data.

#### 4.3.1. Database Establishing Steps

(1) Decide the purpose for establishing the database. The type of data stored in the database, the purpose of the database, and the information acquired from the database are taken into account.
(2) Decide the required data tables in the database. The type of data tables is considered. The data stored in different data tables should not be repeated; high repetition would waste resources because data are repeatedly stored. For this reason, little information should be repeated in different data tables for the connection.
(3) Decide the required data rows in the data table. A data row refers to the data stored in the database. For instance, data rows for name, telephone number, mail address, ID number, and age are required for the data table of personal information.
(4) Determine the correlations between data tables. A data row between different data tables is regarded as the correlation data. For example, ID number is a connection between a personal information table and a human body temperature data table. When one searches for certain data in the data table of human body temperature, ID number can be a useful reference for querying personal temperature information.
(5) Set a unique value. Some data cannot be repeated so the information in such columns should be set as a unique value. For example, name and birth date could be repeated, while ID number is unique. In this case, some specific value would be set to a unique value so that the system would not make wrong judgments.
(6) Establish the database. For different users to be able to use the database at the same time, two different data tables are established. One is the user data table for personal information, and the other is the data tables of human body temperature and heart rate. Regarding columns, name, ID number, gender, and age are covered in the user data table, and ID number is regarded as the unique value. ID number, data, year, month, day, hour, minute, and second are included in the data table for measuring the data. While searching for individual records of physiological signals, ID number is used for the query to prevent users for accessing the wrong data.

### 4.4. Test of Display Interface

To have the user clearly master personal physiological and environmental conditions on the interface through different systems, we integrate the required functions into one display interface, including the measurement interface for running human body temperature and heart rate, the video monitoring interface, and the environment monitoring interface.

#### 4.4.1. Main Interface

**Figure 5.**
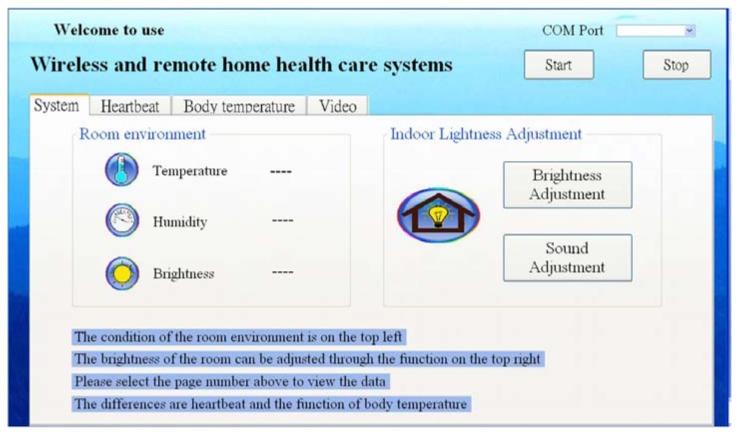
The major interface.

The major interface is as shown as Figure 5. After connecting ZigBee with the computer, the connected COM port could be queried from the device manager. A pull-down menu is offered next to the COM port for looking for the port, and the required data measurement could be selected from the sub-page. ZigBee is connected and starts to transmit data after clicking on Start, while Stop is used for disconnecting ZigBee. When the correct COM port cannot be found in the menu, the operation will be recovered after re-connecting with ZigBee and re-starting the program.

(1) Environment monitoring. Environment monitoring, one of the functions of the interface on the system, would perform the measurements using the temperature, humidity, and brightness sensors equipped on the sensor node in ZigBee, which is placed in the room and instantaneously transmits the data to the computer for the user to observe the current environment in the room.
(2) Environment control. The user could understand the indoor environmental conditions through environmental monitoring and adjust the indoor environment to the most comfortable conditions through environment control. By integrating ZigBee with various electric appliances, this function allows ZigBee to transmitting electric appliance control signals for the user to control the indoor environment through a remote computer.

#### 4.4.2. Temperature Measuring Interface

After the human body temperature data is received, the function would display the current human body temperature till the data are no longer received. Current conditions and the previous exceptions are also displayed. When the temperature is exceptional, the current column would appear as an exception and change the time of the previous exception. After the temperature becomes normal, the exceptional time would remain as the last exceptional time for the user to know the latest physical exceptions. The data measured would be stored in the database anytime, where the past data could be displayed on the system with the query function.

**Figure 6.**
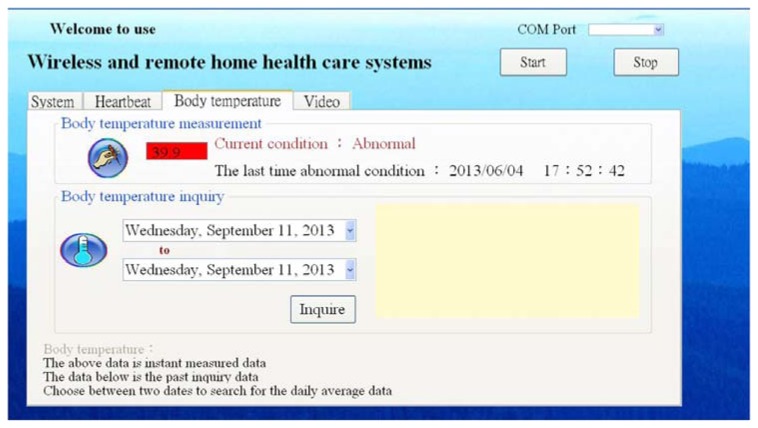
Exceptional human body temperature.

(1) Normal human body temperature. When the human body temperature is normal, the data column would be white, showing the normal state. The last exceptional time records the end time of the latest exception. When the program is just started and no exceptions appear, the last exceptional time would not show any text.
(2) Exceptional human body temperature, as shown as Figure 6. Under exceptional situations, the column appears red and the current state column shows the exception with red text. The previous exceptional time is continuously updated till the state returns to normal.
(3) Human body temperature query. The query function allows acquiring all stored measured data. The query column provides time options for selecting the beginning and ending dates. The program then starts calculating the mean of daily temperature and displays it with graphs on the display for the user clearly showing the daily condition.

#### 4.4.3. Heart Rate Measurement Interface

The self-designed heart rate measurement interface is used for receiving data. The column functions and the interface operation are introduced as below:

(1) Heart rate measurement timing. When the first data are received, the meter starts timing till the measuring equipment is stopped or changed. The signals in the heart rate sensor module are unstable at the beginning for the first 5–10 s as it is not receiving signals. The time in seconds and the heart rate numbers are stored in the database after the signals become stable.
(2) Heart rate values. The measured heart rate signals are transmitted with 1 s and 0 s. The signal from 0 to 1 and back to 0 is regarded as one heartbeat. In this case, one heartbeat is added when the sensing data appears as 1. The column shows the sum of heartbeats till “Stop” is clicked.
(3) Heart rate prediction per minute. When the heart rate is measured and calculated, the estimated value per minute would be calculated. The column would predict the total number of heartbeats per minute. The column is designed as an alert system, allowing the user to find out the personal conditions when the heart rate shows exceptions. When the heart rate per minute exceeds the standard value limit, the system would remind the user with a text alert, showing the current conditions on the system.
(4) Heart rate risk reminder. When the predicted heart rate per minute is too high or too low, the alert system is started to remind the user with red texts. When the heart rate is exceptional, the text would show the current heart rate as being too high or too low.
(5) Heart rate query. The query is similar to the human body temperature query. The beginning and ending dates are first selected, and then the daily situations are displayed with graphs by Chart. The data storage and display are shown every 10 s, and the predicted heart rate per minute is stored every 10 s.

#### 4.4.4. Visual Interface

**Figure 7.**
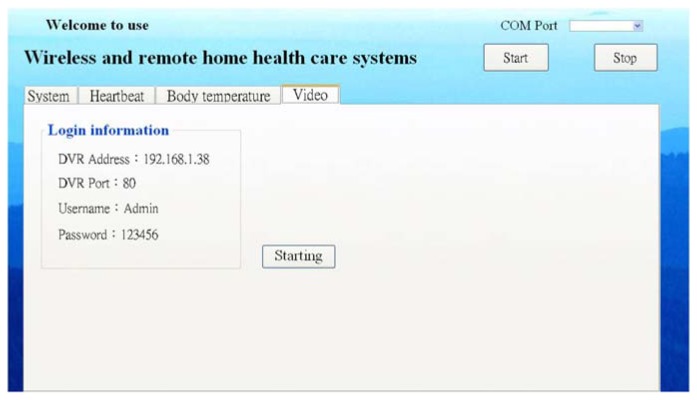
Visual interface.

A connection for the user to fill in the data of IP, COM port, user name, and password to the video is provided on the visual interface, as shown as Figure 7. When the video is started, the above data should be filled in for the frame.

After starting the video and keying in the log-in information, the camera is directly connected to acquire images. Two function tables are displayed after finishing log-in. One is the function table on the bottom-right, and the other is called by clicking on the right button of mouse. The first function table contains quality, screen size, recording, shooting, storage location settings, and zoom, which could be used for taking pictures while viewing images. The second function table provides settings, video on/off, replay, display settings, full screen, and quad-split screens. The display settings include pause, picture-in-picture, zoom out, skip, and lock. The sub-settings functions consist of main menu, search settings, backup, and the Pan Tilt Zoom (PTZ) control. The main menu covers some basic settings of the network, account, camera, and events. Search settings include event search, which could record the time logging in/out the camera account. Time search allows viewing the previously recorded images.

### 4.5. System Analysis

The system is compared with the applications of temperature sensors combined with ZigBee, including a study on nursing care centers [24], wireless tele-homecare systems in [13], and wireless health monitoring system in [14]. The compared items are shown in Table 2.

(1) Platform interface. Regarding the construction of the system platform, both this system and that in [24] are designed with Visual Studio in Microsoft Visual Studio .NET. A new 2008 version was utilized for this system, while 2003 version was used for [24]; C# was applied in [13].
(2) Real-time alerts. Different systems would present distinct real-time alerts; some would inform by e-mail, some others might send mobile messages, and the others might directly make a sound or report to hospitals. In [24], the alert is sent out by sound to remind nurses of exceptional physiological signals of patients. In [13], the reminder on the interface and emergency message are sent to the family's mobile through the SKYPE API. In [14], the alert is reported through the Internet. In addition to providing text alerts on the interface, the alert is sent back through ZigBee in the proposed system for making a sound on the user end to offer real-time advice.
(3) Data view. In regard to the storage in the database, Access is used in [13] and this system selects Microsoft SQL Server. The storage is similar, including time and data, but a query function is also provided in this system. The data in [14,24] appear as data, while the data in this system and [13] are shown with graphs, which offer users useful information.
(4) Remote monitoring. These three systems established a remote monitoring system through a ZigBee wireless network, but the measured physiological signals are slightly different. Human body temperature is measured in [24], human body temperature, blood pressure, and blood oxygen are measured in [13], and human body temperature, heart rate, breathing, and blood oxygen are measured in [14]. The proposed system measures the signals of human body temperature and heart rate.
(5) Environment monitoring. To make the user's living environment more comfortable, this system includes an environment monitoring function. A sensor chip, which transmits data to the computer-based system through ZigBee, is placed in the user's room. The user could determine the indoor conditions through the information on the computer-based system. Furthermore, the integration of the system and various electric appliances could adjust the indoor environment to be more comfortable.
(6) Video system. Being connected with the computer, net-based recorder, and cameras, the real-time images of the cared could be viewed when necessary. The recorder would automatically store all images for the user and the family's queries. The video function allows the family a better understanding of the current state of the user for further concern.

The proposed system contains a wireless transmission module, emergency alert, database analysis and video module. The wireless transmission module utilizes the ZigBee technology. Compared with Bluetooth-based systems, which similarly run for dedicated short-range communications system applications, the proposed ZigBee-based system reaches seven times the transmission distance, increases the number of distributable nodes and reduces the rate of power consumption by 33.5%. Though the transmission speed dropped by 4-fodlld due to lower power consumption, the battery reliability is considered more important than transmission speed for the applications of sensors to tele-healthcare, and the transmission speed that the ZigBee technology reaches is already sufficient for transmitting the amount of vital sign data.

In addition to establishing the tele-healthcare system, the video function is also integrated into the proposed system, so that the pictures of the cared can be transmitted in real time to the medical personnel, which largely enhances the efficiency and quality of healthcare. The principle and the structure are based on the connection among the nursing station, the patient, and the family to integrate an interactive model. With the bedside healthcare video system, the caregiver could directly understand any emergency of the cared through the video frames. The combination of a tele-healthcare system allows the physiological conditions of the cared to be monitored all day long. The prototype of the bedside healthcare video system is designed as a receiving server at the caregiver end, a video camera on the wall next to the bed of the cared, a monitor in front of the bed, a call button, and the physiological monitoring devices, mutually transmitting the information through the wireless sensing network. As improper use of video cameras might infringe the privacy of the cared, for this reason, the video system could be automatically turned on/off to cover the video cameras. Such a cover would merely be opened when there are exceptional physiological data. The privacy of the cared therefore would not be infringed.

The proposed environmental monitoring and the video functions are designed for emergencies, when the real-time dynamic image of the cared could be transmitted to the medical personnel through the video function for immediate and accurate management. Meanwhile, in consideration of privacy, the video camera would be closed and hidden in the wall or a box when there is no emergency or such a function is not necessary. It would be turned on when necessary, in order to protect the privacy in the room. Moreover, when the environment is large, the caregiver end could adjust the angle of camera to the appropriate position and the current situation of the cared when the sensor on the cared or the environmental monitoring system shows exceptions. Since the situation of the cared cannot be actually known from the exceptional physiological data sometimes, such as a sudden increase or decrease of heart rates, the monitoring system would transmit such exceptional physiological data and the medical personnel could rapidly understand the factors in the exceptions through the video frame and procede appropriately. When the user requires medical care because of an accident, the voice and video system established in this study would turn on the video camera for the nurses in the nursing station to rapidly determine the current situations and dealing with the situation in time.

## 5. Conclusions

Constructing a complete homecare monitoring system allows measuring the physiological conditions of the elderly with easy operations, so they do not need to go to hospitals or some specific locations for examination or spend high personnel costs on nurses. They can do the examination at home and transmit the data measured to the computer through wireless transmission. The computer is combined with the database to analyze all data so that the elderly can query the previously measured data on the computer; the living environment of the elderly could be monitored through the system control; the indoor environment could be adjusted to the most comfortable condition by integrating with various electric appliances. The family could also observe the physical conditions of the elderly through the data on the system in real time. In this study, a ZigBee wireless network is utilized for the user to measure the physiological signals anytime and anywhere; the exceptional data could be sent to the user at the first instance; the integration of a database allows the user to study the past physical conditions; the environment monitoring is included for monitoring and adjusting the user's living environment. Regarding the future development, more measuring equipment and sensors could be installed at home to reinforce the physiological monitoring of the user and enhance the living security. The combination of positioning systems could provide the search function, and the integration with hospitals could increase the data availability for the diagnoses of medical personnel.

## Figures and Tables

**Table 1.** Comparison of wireless transmission models.

**Standard**	**WiMAX**	**Bluetooth**	**Wi-Fi**	**ZigBee**
Application	Mobile wireless broadband	Voice/data transmission/appliance accessories	Voice/data/audio/video transmission	Monitoring/data transmission/control sensor
Frequency band	10/20 MHz	2.4 GHz	2.4 GHz	868 MHz/915 MHz/2.45 GHz
Transmission rate	30/75 Mbps	2–3 Mbps	11 Mbps	20/40/250 Kbps
Safety	Low	High	Low	Medium
Distance	5–7 KM	10 M	100 M	100–400 M
Number of network nodes	N/A	8	127/HOST	65536

**Table 2.** Analysis of system function.

**System Function**	**The Proposed Healthcare System**	[24]	[13]	[14]
Platform interface	Yes	Yes	Yes	Yes
Real-time alert	Yes	Yes	Yes	Yes
Data view	Yes	Yes	Yes	Yes
Remote monitoring	Human body temperature, heart rate	Human body temperature, heart rate, breathing, blood oxygen	Human body temperature	Human body temperature, blood oxygen, blood pressure
Environment monitoring	Yes	No	No	No
Video system	Yes	No	No	No
